# Safety and Technological Characterization of *Staphylococcus xylosus* and *Staphylococcus pseudoxylosus* Isolates from Fermented Soybean Foods of Korea

**DOI:** 10.4014/jmb.2111.11040

**Published:** 2022-01-02

**Authors:** Haram Kong, Do-Won Jeong, Namwon Kim, Sugyeong Lee, Sooyoung Sul, Jong-Hoon Lee

**Affiliations:** 1 Department of Food Science and Biotechnology, Kyonggi University, Suwon 16227, Republic of Korea; 2 Department of Food and Nutrition, Dongduk Women’s University, Seoul 02748, Republic of Korea; 3 Division of Sports Science, Kyonggi University, Suwon 16227, Republic of Korea

**Keywords:** Fermented soybean, *Staphylococcus xylosus*, *Staphylococcus pseudoxylosus*, antibiotic resistance, salt tolerance, protease activity

## Abstract

We evaluated the antibiotic susceptibilities, hemolytic activities, and technological properties of 36 *Staphylococcus xylosus* strains and 49 *S. pseudoxylosus* strains predominantly isolated from fermented soybean foods from Korea. Most of the strains were sensitive to chloramphenicol, erythromycin, gentamycin, kanamycin, lincomycin, oxacillin, tetracycline, and trimethoprim. However, 23 strains exhibited potential phenotypic acquired resistance to erythromycin, lincomycin, and tetracycline. Based on breakpoint values for staphylococci from the Clinical and Laboratory Standards Institute, >30% of the isolates were resistant to ampicillin and penicillin G, but the population distributions in minimum inhibitory concentration tests were clearly different from those expected for acquired resistance. None of the strains exhibited clear α- or β-hemolytic activity. *S. xylosus* and *S. pseudoxylosus* exhibited salt tolerance on agar medium containing 20% and 22% (w/v) NaCl, respectively. *S. xylosus* and *S. pseudoxylosus* strains possessed protease and lipase activities, which were affected by the NaCl concentration. Protease activity of *S. pseudoxylosus* was strain-specific, but lipase activity might be a characteristic of both species. This study confirms the potential of both species for use in high-salt soybean fermentation, but the safety and technological properties of strains must be determined to select suitable starter candidates.

## Introduction

Coagulase-negative staphylococci (CNS) are part of the normal microbiota of the skin and mucous membranes of humans and animals, and they are also distributed in a variety of niches including soil, water, and air, as well as various foodstuffs [1, 2]. They have been identified as prevalent members of the beneficial microbiota in animal-derived fermented foods; in particular, their technological properties have been well characterized in meat fermentation. CNS have been reported to contribute to the development and stability of product color through their nitrate reductase activity, and flavor development through protease and lipase activities [3].

Several types of plant-derived fermented foods are consumed in Korea; kimchi (the generic term given to fermented vegetables) and jang (the generic term given to fermented soybean foods) are representative examples [4, 5]. The representative types of jang are ganjang (soybean sauce) and doenjang (soybean paste). Several studies that included microbial community analysis have been performed to provide basic insight for accelerated ripening, quality assurance, and flavor enhancement of traditional Korean fermented foods. Early microbial studies isolated and identified microorganisms exhibiting target technological properties including high enzyme activities that contribute to the fermentation process [6-9]. Recently, the advent of culture-independent microbial community analysis techniques has revealed the presence of a wider variety of microorganisms in the food matrices [10-14]. CNS are one of the bacterial groups whose prevalence in fermented soybean foods from Korea was identified by the implementation of culture-independent microbial community analysis techniques.

Traditional manufacture of ganjang and doenjang starts from meju. Meju is made by soaking, steaming, crushing, and molding soybeans, which then naturally ferment for 2–3 months. Ripened meju is used as a starter culture as well as a nutrient and flavor source for the production of several types of jang, including ganjang and doenjang [5, 15]. The ripened meju is mixed with brine and ripened for a further 2–3 months, then the liquid portion of the mixture is separated, resulting in a traditional type of ganjang. The remaining solid portion is subsequently mashed and fermented for >6 months and becomes doenjang.

Our previous study performed with several types of culture media supplemented with NaCl enabled the isolation of CNS in the ripening process of doenjang; *Staphylococcus saprophyticus* was identified as the predominant species among the CNS [16]. In a subsequent culture-dependent study [17] for the identification of the predominant bacterial species in meju using 12 meju samples collected from five regions of Korea, six species of CNS were identified, and *S. xylosus* was the most populous species. Their prevalence in fermented soybean foods indicates the potential of *S. saprophyticus* and *S. xylosus* as starter culture candidates for soybean food fermentation.

Meanwhile, recent progress in phylogenomic analysis has consolidated and differentiated closely-related *Staphylococcus* species and a novel taxon, *S. pseudoxylosus*, has been introduced [18]. The 16S rRNA gene sequence of *S. pseudoxylosus* is similar to that of *S. saprophyticus*, *S. caeli*, *S. edaphicus* and *S. xylosus*.

In this study, we first confirmed the taxonomic status of our stock *S. xylosus* and *S. saprophyticus* strains isolated from meju and doenjang [16, 17]. We then determined their antibiotic resistance profiles, hemolytic activity, and technological characteristics to assess their potential of introduction in soybean food fermentation.

## Materials and Methods

### Strains, Cultures, and Taxonomic Identity Confirmation

Fifty-one strains previously classified as *S. xylosus* and 34 as *S. saprophyticus* by near-complete 16S rRNA gene sequence analysis were used in this study. These strains were isolated from meju and doenjang [16, 17]. Here, the identity of the isolates was confirmed by analyses of the sequences of the housekeeping genes *gmk* (encoding guanylate kinase) and *gap* (encoding glyceraldehyde 3-phosphate dehydrogenase). The PCR primer sets used in the amplification of *gmk* and *gap* were: *gmk* forward, 5′-GAC AAG CGT GAA GGT GAA GTC-3′; *gmk* reverse, 5′-GTT CAT CAT TTC AAC TTC TCG-3′; *gap* forward, 5′-CTG AAA CAA TTG CTC ACC-3′; and *gap* reverse, 5′-GCA GCA CCT GTA GAA GTT GG-3′. The PCR conditions used for amplification of *gmk* and *gap* were the same as those in our previous studies [16, 17]. *S. xylosus* S170 was kindly provided by the Rural Development Administration of Korea [19]. *S. xylosus* SMQ-121, a commercially available meat starter, was purchased from the Universite Laval, Canada [20]. Strains were cultured in Difco tryptic soy agar (TSA; BD Diagnostic Systems, USA) and Difco tryptic soy broth (TSB; BD Diagnostic Systems) at 37°C for 24 h.

### Determination of Minimum Inhibitory Concentrations (MICs)

MICs of antibiotics were determined according to the guidelines of the Clinical and Laboratory Standards Institute (CLSI) [21] by the broth microdilution method. Ten antibiotics (ampicillin, chloramphenicol, erythromycin, gentamycin, kanamycin, lincomycin, oxacillin, penicillin G, tetracycline, and trimethoprim) frequently used to assess antibiotic resistance of CNS were used in this study [22-25]. Up-to-date breakpoint values for staphylococci provided by the CLSI (2021) [26], the European Committee on Antimicrobial Susceptibility Testing (EUCAST, 2021) [27], the United States Committee on Antimicrobial Susceptibility Testing (USCAST, 2021) [28], and the Comité de l’Antibiogramme de la Société Française de Microbiologie (CA-SFM, 2021) [29] were adopted to evaluate susceptibility to each antibiotic (Table 1). In the case of ampicillin and kanamycin, the most recent breakpoints of the CLSI were used (2010 and 2017, respectively) [30, 31]. A two-fold serial dilution was prepared for each antibiotic in deionized water, and the final concentrations in each well of a microplate ranged from 0.03 to 512 mg/l. Test strains were cultured twice in TSB and matched to a 0.5 McFarland turbidity standard (bioMérieux, France). The cultured strains were diluted (1:100) in Difco Mueller-Hinton broth (BD Diagnostic Systems) to achieve the desired inoculum concentration. The final inoculum density in each well was 5×10^5^ colony forming units/ml. The microplates were incubated at 37°C for 18 h. The MIC of each antibiotic was determined as the lowest concentration at which no turbidity was observed in the wells. The MIC tests were performed in triplicate.

### Hemolytic Activity Test

α-Hemolytic activity was determined on TSA containing 5% (v/v) rabbit blood (MB Cell, Korea). β-Hemolytic activity was determined on TSA containing 5% (v/v) sheep blood (MB Cell). α-Hemolytic activity was determined by incubation at 37°C for 24 h, and β-hemolytic activity was determined by incubation at 37°C for 24 h and cold shock at 4°C for 24 h. *S. aureus* Newman was used as a positive control for the hemolytic analysis [22, 32]. All the experiments were performed at least three times on separate days.

### Assessment of Technological Characteristics of Strains

The salt tolerance of CNS strains was determined by growth on TSA supplemented with NaCl [final concentration 19%–24% (w/v)] with incubation at 37°C for 4 days. Protease activity was determined on TSA containing 2% skim milk (w/v). Lipase activity was determined on tributyrin agar (Sigma-Aldrich, USA) containing 1% tributyrin (v/v). Enzyme activity was determined from the clear zone developed after incubation at 37°C for 4 days. The effect of NaCl concentration on protease and lipase activities was determined by addition of NaCl to the activity test media. The test strains were incubated in TSB to optical density 0.5 at 600 nm and 1 μl of this suspension was inoculated onto each plate. Experiments were performed at least three times on separate days.

## Results

### Taxonomic Status of CNS Strains

Sequence analysis of the genes *gmk* and *gap* indicated that our stock cultures included 36 *S. xylosus* and 49 *S. pseudoxylosus* strains.

### Prevalence of Phenotypic Antibiotic Resistance

Table 1 shows the MICs of the test antibiotics toward the 36 *S. xylosus* and 49 *S. pseudoxylosus* strains. Most of the strains were sensitive to eight antibiotics (chloramphenicol, erythromycin, gentamycin, kanamycin, lincomycin, oxacillin, tetracycline, and trimethoprim). However, based on the 2010 CLSI breakpoint value for ampicillin for staphylococci, 33.3% and 30.6% of the *S. xylosus* and *S. pseudoxylosus* strains, respectively showed ampicillin resistance [30]. When the 2021 standard of the CLSI was applied, 97.2% and 65.3% of the *S. xylosus* and *S. pseudoxylosus* strains, respectively, were resistant to penicillin G [26]. Note that the breakpoint values suggested by the four organizations differ, and thus application of the lowest breakpoint values increases the number of resistant strains. Two *S. xylosus* strains exhibited high resistance to erythromycin; high lincomycin resistance was identified in eight *S. xylosus* strains and two *S. pseudoxylosus* strains; eight *S. xylosus* strains and four *S. pseudoxylosus* strains exhibited high tetracycline resistance. Their resistance to erythromycin, lincomycin, and tetracycline was >32-fold higher than the corresponding breakpoints specified by the EUCAST, USCAST, and CA-SFM [27-29]. The population distributions of the two species in MIC tests with erythromycin, lincomycin, and tetracycline were bimodal, which can be considered a phenotypic acquired antibiotic resistance profile [33]. Among the 23 strains showing patterns of phenotypic acquired resistance, one strain of *S. xylosus* exhibited resistance to erythromycin and tetracycline (data not shown).

### Hemolytic Activity

**Fig. 1:**
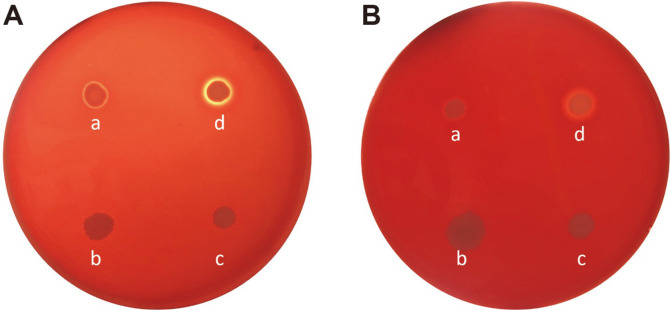
α-Hemolytic (A) and β-hemolytic (B) activity of *Staphylococcus pseudoxylosus* strain 14AME19. Strains: a, *S. pseudoxylosus* 14AME19; b, *S. xylosus* S170; c, *S. xylosus* SMQ-121; d, *S. aureus* Newman (positive control).

Hemolytic activity was tested in the 36 *S. xylosus* strains and 49 *S. pseudoxylosus* strains. *S. pseudoxylosus* strain 14AME19 exhibited weak α- and β-hemolytic activities (Fig. 1). None of the other strains showed α- or β-hemolytic activity.

### Technological Properties of Strains

**Fig. 2:**
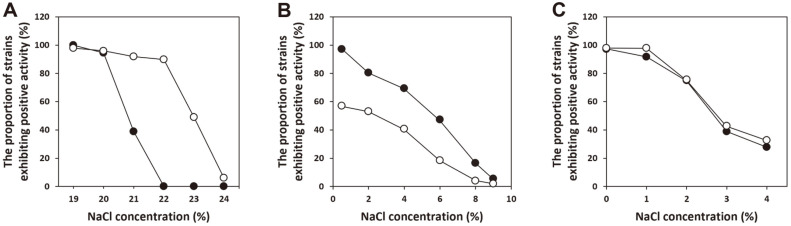
Technological properties of 36 *S. xylosus* strains (●) and 49 *S. pseudoxylosus* strains (○). Salt tolerance (**A**), protease activity (**B**), and lipase activity (**C**) were evaluated on agar plates supplemented with NaCl (concentrations in % w/v).

Over 90% of the *S. xylosus* and *S. pseudoxylosus* strains grew on TSA plates containing 20% and 22% NaCl (w/v), but not at NaCl concentrations of 22% and 24%, respectively (Fig. 2). Most *S. xylosus* strains (97.2%) exhibited protease activity on the activity test plates containing 0.5% NaCl, but 42.9% of *S. pseudoxylosus* strains did not. Nearly all the *S. xylosus* and *S. pseudoxylosus* strains lost protease activity when the NaCl concentration was increased to 9% (w/v). Over 97% of the *S. xylosus* and *S. pseudoxylosus* strains exhibited lipase activity on activity test plates, but the number of lipase-positive strains of both species decreased in similar fashion with increasing NaCl concentration; the proportion of *S. xylosus* and *S. pseudoxylosus* strains positive for lipolytic activity was 27.8% and 32.7%, respectively, at 4% (w/v) NaCl.

## Discussion

Several types of fermented soybean food are consumed in East Asia; they have drawn the attention of food scientists who wish to unravel the roles of microorganisms in the flavor and quality of products from each region [34]. Culture-independent approaches have revealed the dynamics of microbial communities in food matrices and contributed to revealing CNS as a predominant bacterial group in Korean fermented soybean foods. Meanwhile, microbial community analysis based on culture methods to develop starters for animal-derived fermented foods proved the existence of several CNS species, including *S. xylosus*, *S. equorum*, *S. carnosus*, *S. saprophyticus*, *S. warneri*, *S. epidermidis*, *S. pasteuri*, and *S. succinus* in the matrices [35-38].

A number of molecular methods for rapid and reliable species-level identification of CNS have been used in the course of microbial community analysis. Methods based on 16S rRNA gene sequences are generally adopted for bacterial identification, but are well known to exhibit low accuracy among members of a given genus or species because of the high degree of sequence similarity [39]. To overcome this limitation, alternative target genes, including *rpoA*, *rpoB*, *sodA*, *tuf*, and *hsp60*, have been evaluated for CNS identification [39-41]. In the present research, we chose the sequences of *gmk* and *gap* to confirm the taxonomic status of *S. xylosus* and *S. saprophyticus* strains; sequences of these genes were suitable for differentiating *S. xylosus* and its close relatives. The sequence of *gap* was confirmed to show the highest resolving power among the reported target genes used for CNS species detection (Table 2). Through the reidentification of our stock cultures of CNS isolates based on the current taxonomic classification, *S. pseudoxylosus*, not *S. saprophyticus*, was shown to be the predominant CNS species in meju and doenjang, together with *S. xylosus*.

This study has been the first to assess the antibiotic susceptibilities of *S. pseudoxylosus* strains isolated from fermented soybean foods. Our MIC test results confirmed that the breakpoint values for staphylococci provided by the CLSI (2021) [26] can be applied to Korean *S. xylosus* and *S. pseudoxylosus* isolates to test for antibiotic susceptibility in the case of chloramphenicol; those of EUCAST (2021) [27] are applicable for erythromycin, gentamycin, oxacillin, tetracycline, and trimethoprim; and those of CA-SFM (2021) [29] are applicable for kanamycin and lincomycin. When the applicable breakpoint values were adopted for antibiotic susceptibility evaluation, a few of our strains were found to be resistant to oxacillin and trimethoprim. However, these resistances were not high enough (<4-fold the applicable breakpoint values) to consider the strains as resistant, but they may be considered specific characteristics of the strains [33]. Meanwhile, >30% of the isolates exhibited resistance to ampicillin and penicillin G when the breakpoints of the CLSI (2010 and 2021, respectively) [26, 30] were applied. High percentage occurrence of ampicillin and penicillin resistant strains was also observed in *S. xylosus* isolates from meat products by using microdilution assay [42]. The population distributions of the two species in MIC tests with ampicillin and penicillin G were continuous or unimodal. The profiles were clearly different from those determined with erythromycin, lincomycin, and tetracycline (acquired resistance profiles). Thus, the breakpoint values for ampicillin and penicillin G suggested for staphylococci by the CLSI (2010, 2021)[26, 30] need to be reconsidered for *S. xylosus* and *S. pseudoxylosus* through further studies. The identification of strains exhibiting potential phenotypic acquired antibiotic resistance to erythromycin, lincomycin, and tetracycline highlights the necessity for antibiotic susceptibility testing before food application. The higher prevalence of potential phenotypic acquired resistance among strains of *S. xylosus* compared with *S. pseudoxylosus* requires studies of the antibiotic-resistance gene acquisition of both species.

The results of phenotypic hemolysis tests indicate that *S. xylosus* and *S. pseudoxylosus* isolates from Korea do not present a high risk in terms of hemolytic activity, but the observation of one strain with weak hemolytic activity requires hemolytic activity tests for starter development. Genomic analysis of *S. pseudoxylosus* strain 14AME19, the strain with some hemolytic activity (Fig. 1), showed that its genome does not contain any α-hemolysin, β-hemolysin, or enterotoxin gene homologs characteristic of pathogenic *S. aureus* [43]. The weak hemolytic activity of strain 14AME19 was attributed to its strong lipolytic activity that led to degradation of erythrocyte membrane phospholipids. The genome of strain 14AME19 includes two annotated triacylglycerol lipase genes and three lysophospholipase genes.

This study is the first to present physiological differences between *S. xylosus* and *S. pseudoxylosus*. *S. pseudoxylosus* exhibited higher salt tolerance than *S. xylosus* in spite of their close phylogenetic relatedness. Growth at an NaCl concentration of ≥22% (w/v) can be used as a criterion to differentiate the two species. Strains showing higher salt tolerance than the average tolerance of each species may possess additional salt tolerance determinants [44]. The high salt tolerance of both species is well matched with their prevalence in high-salt fermented foods and can contribute to production of high-salt fermented soybean foods. The proportion of protease-positive *S. xylosus* strains was higher than that of *S. pseudoxylosus*, but the effect of increasing NaCl concentration on the protease activity was similar in both species. Most of the strains possessed lipase activity, and the effect of NaCl was similar regardless of the species. The possession of protease may be strain-specific in *S. pseudoxylosus*, but possession of lipase may be a characteristic of both species. The effect of NaCl on the protease and lipase activities of cells may be attributed to the characteristics of the relevant enzymes or physiological changes in cells that influence the enzyme production.

This study confirmed that *S. pseudoxylosus*, the closest known relative of *S. xylosus*, does not exhibit distinguishable differences with *S. xylosus* in terms of antibiotic resistance or hemolytic activity. *S. xylosus* has been the most frequently isolated CNS species and it is used in commercial cultures in European meat fermentation [45]. The Ministry of Food and Drug Safety of Korea (https://www.mfds.go.kr/) has allowed its use only in meat fermentation. The history of the use of *S. xylosus* in meat fermentation may positively influence the introduction of *S. xylosus* and *S. pseudoxylosus* in soybean fermentation. However, the European Food Safety Authority (EFSA) has not given *S. xylosus* Qualified Presumption of Safety (QPS) status as of 2020 [46]. The absence of *S. xylosus* from the QPS status list may be a big obstacle to the use of *S. pseudoxylosus* and *S. xylosus* in soybean food fermentation because the safety of metabolites produced by *S. xylosus* in such fermentation has not been proved. Further genomic and metabolomic studies to illuminate the safety of both species will determine their destiny in East Asian food fermentation.

## Figures and Tables

**Table 1:** Distribution of 36 *Staphylococcus xylosus* and 49 *S. pseudoxylosus* strains isolated from fermented soybean foods from Korea over a range of minimum inhibitory concentrations (MICs) for ten antibiotics.

Antibiotic	Species	MIC (mg/l)															Breakpoint (mg/l)			
	
		0.03	0.06	0.13	0.25	0.5	1	2	4	8	16	32	64	128	256	512	CLSI	EUCAST	USCAST	CA-SFM
Ampicillin	*S. xylosus*			1	23	12											R ≥ 0.5	–	–	–
	*S. pseudoxylosus*		4	13	17	12	3										(2010)			
Chloramphenicol	*S. xylosus*					1	1	1	6	21	6						R ≥ 32	R > 8	–	R > 8
	*S. pseudoxylosus*								1	30	18									
Erythromycin	*S. xylosus*					22	11	1						2			R ≥ 8	R > 2	R ≥ 4	R > 2
	*S. pseudoxylosus*					26	17	6												
Gentamycin	*S. xylosus*					36											R ≥ 16	R > 1	R ≥ 2	R > 1
	*S. pseudoxylosus*					48	1													
Kanamycin	*S. xylosus*					30	4	1		1							R ≥ 64	–	–	R > 8
	*S. pseudoxylosus*					41		7	1								(2017)			
Lincomycin	*S. xylosus*						1	1	11	15						8	–	–	–	R > 8
	*S. pseudoxylosus*									47						2				
Oxacillin	*S. xylosus*	1	5	3	26	1											R ≥ 1	R > 0.25	R > 0.25	R > 0.25
	*S. pseudoxylosus*		8	7	31	2	1													
Penicillin G	*S. xylosus*			1	18	16	1										R ≥ 0.25	–	–	–
	*S. pseudoxylosus*	2	5	10	10	19	2	1												
Tetracycline	*S. xylosus*					28							1	6	1		R ≥ 16	R > 2	R ≥ 4	R > 2
	*S. pseudoxylosus*					45								4						
Trimethoprim	*S. xylosus*					3	12	14	6	1							R ≥ 16	R > 4	R ≥ 8	R > 4
	*S. pseudoxylosus*					1	1	28	18	1										

Breakpoint values for staphylococci provided by the Clinical and Laboratory Standards Institute (CLSI, 2021) [26], the European Committee on Antimicrobial Susceptibility Testing (EUCAST, 2021) [27], the United States Committee on Antimicrobial Susceptibility Testing (USCAST, 2021) [28], and the Comité de l’Antibiogramme de la Société Française de Microbiologie (CA-SFM, 2021) [29] were adopted to evaluate susceptibility to each antibiotic. For ampicillin and kanamycin, the breakpoints of CLSI published in 2010 and 2017 were used, respectively [30, 31].

**Table 2:** Nucleotide sequence similarities (%) of genes between *Staphylococcus xylosus* and its relatives.

Species	Gene							

	16S rRNA	*rpoA*	*sodA*	*tuf*	*rpoB*	*hsp60*	*gmk*	*gap*
*S. pseudoxylosus* S04009^T^	99.8	98.8	98.7	98.6	98.0	96.9	96.2	93.6
*S. saprophyticus* subsp. *saprophyticus* NCTC13634^T^	99.8	95.3	94.7	97.1	93.7	88.4	89.4	82.7
*S. caeli* 82B^T^	99.7	95.2	93.7	96.6	91.9	88.3	89.0	82.8
*S. succinus* 14BME20	99.4	94.3	92.5	94.6	91.0	86.1	87.7	81.3
*S. equorum* subsp. *equorum* NCTC12414^T^	99.1	93.1	93.3	91.6	92.5	88.6	87.8	82.6
*S. warneri* NCTC11044^T^	98.4	89.2	86.4	90.9	87.4	83.3	80.0	75.2
*S. pasteuri* SP1	98.3	89.5	86.9	90.8	87.4	84.1	80.3	75.9
*S. epidermidis* NBRC100911^T^	97.9	90.1	85.5	91.7	85.4	80.8	76.5	77.2
*S. carnosus* subsp. *carnosus* TM300	97.2	88.7	82.9	91.8	84.4	81.7	78.9	74.6

The genes of *S. xylosus* ATCC 29971^T^ were used as the reference for nucleotide sequence comparisons.

Genes: 16S rRNA, 16S ribosomal RNA; *rpoA*, DNA-directed RNA polymerase subunit alpha; *sodA*, superoxide dismutase; *tuf*, elongation factor Tu; *rpoB*, DNA-directed RNA polymerase subunit beta; *hsp60*, heat-shock protein 60; *gmk*, guanylate kinase; *gap*, glyceraldehyde 3-phosphate dehydrogenase.
